# Social support as a protective factor for children impacted by HIV/AIDS across varying living environments in southern Africa

**DOI:** 10.1080/09540121.2016.1176683

**Published:** 2016-07-08

**Authors:** Edna Barenbaum, Tamarah Smith

**Affiliations:** ^a^ Department of Psychology, Cabrini College, Radnor, PA, USA

**Keywords:** HIV, AIDS, orphans, caregiver, psychological well-being, social support

## Abstract

The literature on the psychological well-being of children impacted by HIV/AIDS in Africa highlights increased vulnerability due to loss of parents and environmental stressors (e.g., hunger). Research shows that the lack of attachment and social support due to loss limits the grieving process in children. Access to trusting adults and social support through caregivers can be an important protective factor to allow for coping and better emotional adjustment in the future. This study examined social support systems across varying living environments to determine if social support promoted higher levels of well-being in children orphaned and made vulnerable by HIV/AIDS. The participants included 100 children from a small targeted population in southern Africa who receive varying levels of support from a private not-for-profit organization. Children’s well-being was assessed through the Psycho-Social Adjustment Scale-Adolescents developed specifically for vulnerable child populations in Africa. Children were individually interviewed either on their homestead, school or hostel. Data demonstrated that children who do not share their feelings had significantly lower measures of positive well-being (*M *= 2.61 (0.87) vs. *M *= 3.10 (0.57), *d *= 0.60). Children with trusted adults were significantly more likely to share their feelings and had lower incidence of hunger (49.1% vs. 62.5%), suicide ideation (15.1% vs. 62.5%) and witnessing violence (69.8% vs. 87.5%). Sharing feelings with caregivers was more pronounced among children who had greater access to trusted adults and correlated with stronger attachment scores (*r *= .30, *p *< .01). An important component to decrease levels of anxiety and depression in this vulnerable population is providing access to trusted individuals. Social support interventions are discussed.

Through a developmental psychopathological approach, one strives to understand the complexity of human development as a dynamic transaction between the person and the environment. Additionally, developmental psychopathology is interested in the range of outcomes from normal development to abnormal development and in-between. The multiple outcomes on this continuum are consistent with social ecological theory (Bronfenbrenner, 1979) and are a result of risks or protective factors due to various environmental settings, family or other persons with whom an individual may interact. A developmental psychopathological approach recognizes that behavior can change and is not static allowing for individual and environmental conditions which are also constantly changing overtime. One of the most significant factors that affect an individual’s development along the developmental continuum is bereavement, which in turn impacts psychosocial development over a lifetime.

## Bereavement

Bereavement due to the death of a parent during childhood can have a profound and lifelong impact on a child’s psychosocial well-being. Research has largely neglected to investigate the psychological effects of parental death within the AIDS epidemic (Li et al., 2008; Sherr & Mueller, 2008). Cross-cultural research on natural grieving processes suggests that most humans need to recognize their grief and be able to express it in order to resolve their loss (Fraley & Shaver, 1999). Children are at increased risk for unresolved or complicated bereavement due to their developmental vulnerability, such as their intellectual immaturity and emotional dependency (Li et al., 2008). Children orphaned by AIDS may face additional psychological and social challenges impacting their well-being including stigmatization, the impending or actual death of the surviving parent, disruptions in subsequent care and financial hardships. Sherr and Mueller (2008) point out that HIV and AIDS may directly disrupt many of the protective mechanisms identified in the general bereavement literature. For example, the fact that HIV is sexually transmitted means that disease and death clusters in families, thereby compromising subsequent caregiving. Many of the children orphaned by HIV/AIDS are surrounded by poverty, face disruptions and lack of social support services, and often experience caregiver and residential instability following traumatic illness and death from AIDS. Cluver and Gardner (2007) found that children’s psychological well-being was affected by homelessness, migration, unequal treatment, limited resources, stigma and multiple bereavement of the household heads by HIV and AIDS. Wood, Chase, and Aggleton (2006) also suggest that vulnerability of children created by parental/familial death lies on a continuum, where it increases as more family members die. These challenges may further impede the grieving process placing these children at heightened risk of prolonged mental and behavioral problems (Cluver & Gardner, 2006; Sachs & Sachs, 2004). It is unfortunate that limited attention has been paid to the psychosocial and developmental needs of these children made vulnerable and orphaned by HIV/AIDS, particularly in resource-poor countries or regions (Atwine, Cantor-Graae, & Bajunirwe, 2005; Cluver & Gardner, 2007). From 2001 to 2005, the global number of children orphaned by AIDS increased from 11.5 million to 15 million, and 12 million of these AIDS orphans were living in Sub-Saharan Africa (UNICEF, 2004). It is estimated based on current trends that the number of AIDS orphans could reach 40 million by 2020 (UNICEF, 2004).

## Attachment and caregiving as a risk or protective factor

The positive impact of mentors is consistent with other’s arguments that factors that affect the mental health of children orphaned by AIDS include different care arrangements such as home care, kinship care or institutional care and the quality of the caregiver–child relationship (Cluver & Gardner, 2007). Sherr et al. (2014) indicate that population-based surveys in highly affected countries suggest that the vast majority (90%) of orphans live within extended families, whereas 10% live with unrelated caregivers. They suggest that evidence appears to indicate that in highly affected countries, the number of children’s homes (orphanages) is increasing. These contextual factors concern the extent to which caregivers are available both physically and emotionally for a child during bereavement (Bowlby, 1982). Healthy attachment occurs when the child perceives an adult as trusting and, therefore, approachable. Without attachment figures children are at risk for growing up with unrecognized and unaddressed grief resulting in complicated grief with prolonged negative emotions that are often expressed in anger and depression. Lack of interpersonal contact and distant involvement with adults impedes the child’s ability to develop healthy human relationships necessary for acceptance of the loss and life fulfillment. Research based on attachment theory has highlighted the need to recognize the quality of the child’s attachment relationships before and after the loss (Bowlby, 1982). Research has also shown that caregiver sensitivity and emotional availability are key in determining children’s attachment and subsequent security (Chisholm, 1998). When access to caregivers is limited, such as with orphaned and vulnerable children (OVC), these changes in the child’s environment are important as they may influence whether the child has secure attachments (Provence & Lipton, 1962). Previous literature suggests severe developmental consequences when children are placed in institutional care, which affords neither stimulation nor consistent attachment relationships with caregivers. These child-rearing environments are universally assumed to be undesirable, if not pathogenic (Larose, Berneir, & Tarabulsy, 2005). Such experiences are referred to as conditions of “social deprivation” (O’Connor & Rutter, 2000). However, much of this literature is based on orphanage-reared children from Romania adopted and living in the United States, and there has been little to address the affect of changes in caregiving environments among OVC due to HIV/AIDS in Africa.

## Evidence of impact on well-being

Chi and Li (2013) in their substantive review of the impact of parental HIV/AIDS on children’s psychological well-being point out that prior to 1994 there were no published empirical studies that investigated the psychological outcomes and grief reactions of children orphaned by AIDS. By 2001, Wild had identified only six published empirical studies of well-being with only one of them conducted in Africa. However, given the lack of comparison data, these studies provided insufficient information to determine the extent to which losing a parent to AIDS placed children at an increased risk for psychological distress. Wild recommended that future researchers use comparison data to identify the personal, familial and community factors that might account for or influence the relationship between parental HIV/AIDS and children’s psychological well-being.

In 2007, Cluver and Gardner reviewed 24 published and unpublished empirical studies that examined mental health of children orphaned by HIV/AIDS. Sixteen of the studies included comparison groups with 13 of the studies conducted in Africa and 3 studies conducted in the United States. Their review suggested that children orphaned by HIV/AIDS might experience higher levels of psychological difficulties. Additionally, there were more internalized problems than externalized problems among the children orphaned by HIV/AIDS. The authors suggested that orphans, particularly those in Africa, were likely to experience more emotional problems such as depression and anxiety than conduct or other problem behaviors. The authors called for additional rigorous studies with standardized instruments, appropriate comparison groups and large sample sizes.

Chi and Li’s (2013) expanded review of the literature on children affected by HIV/AIDS provided more detail not only on the psychological difficulties faced by these children but also on the potential impact of various risk and protective factors. In total, 30 studies were reported including 18 studies from Africa, 8 from the United States, 1 from Italy and 3 from China. Seven studies included data from children and caregivers as an additional source of information with one study only collecting data from caregivers. Several studies determined that children having adequate coping skills can adjust well with traumatic events including parental illness and death and have a better psychological adjustment. It was suggested that a trusting relationship with current caregivers might be a global protective factor for children’s psychological adjustment in coping with life stressors. Another stress buffer, as found by Onuoha and Munakata (2010) who addressed the perceived or actual social support through a natural mentor (e.g., adults other than caregivers to whom a child can go to for support), showed significantly better psychological well-being than those who did not have one.

## Study aims and goals

Although some studies have examined the role of social support and mentorship (e.g., Onuoha & Munakata, 2010), few published studies have included attachment in the examination of the psychological well-being of children including the attachment of children orphaned by AIDS. Because AIDS often disproportionately affects young and middle-age adults in families, AIDS orphans tend to be younger than other non-AIDS-orphaned children in these environments. The care of AIDS-orphaned children is generally provided by older adults (e.g., grandmother) or in some cases children remain in child-headed households after their families have died. This brings into question if care by the extended family systems or by child-headed households is adequate given the increase in disadvantaged children and severe economic constraints these families experience (Abebe & Aase, 2007). Makame, Ani, and Grantham-McGregor (2002) suggested that children living in child-headed households or with grandparents have the most serious psychological problems because of lack of necessary and adequate social and emotional support in these households.

This study represents a step in comparing the potential impact of varying levels of care among two different living arrangements of OVC in southern Africa by addressing the well-being of the children and their relationship with current caregivers. Psychological well-being as defined by WHO (2001) is “a state of well-being in which the individual realizes his or her own abilities, can cope with the normal stresses of life, can work productively and fruitfully, and is able to make a contribution to his or her community” (p. 2). Chi and Li (2013) addressed well-being “with regard to the children affected by HIV/AIDS, psychological well-being refers to the procession of emotional, behavioral and social competence appropriate to their developmental stages and the resilience in adversity of parental illness and death”. The authors assert that psychological well-being is one of the important elements of children’s health and development. Careful attention to the role of attachment and sharing feelings with trusted adults between groups was measured. It was expected that those children with adequate social support most notably trusted adults with whom they could share feelings, both positive and negative, would have higher levels of positive well-being.

## Methods

### Participants

The participants for the study included 100 children (57% females) from a small targeted population in southern Africa who receive support from a private not-for-profit organization (NFPO). The children were part of one of two living arrangements. The first group considered the most vulnerable children in the population lived in a “hostel” setting receiving comprehensive care from the organization including their shelter, health care, food, clothing and education (43%). This included individuals coming from child-headed homesteads or in instances where the safety of the child was at risk. Children in the second group also received financial support from the same organization, including health care, school supplies and uniforms, but did not require shelter (57%). Typically, they lived with extended family members on a homestead and traveled by foot, to and from their school and home setting. The homestead sample had more females than the hostel sample (53.8% vs. 33.8%) but the groups were similar in terms of age (homestead: *M *= 17.41 (2.33); hostel: *M *= 16.54 (2.11**))**. The children, regardless of residence, were all under the care of the organization given their status as vulnerable or orphaned by HIV/AIDS. To be included in the study, children were required to be proficient in English and available during the time of the researchers’ visit. Every child between the ages of 12 and 22 who resided at or received support from the NFPO were eligible to participate in the study. This consisted of 150 children. Of this, 100 were available during the scheduled visits and fluent in English, and only scheduling conflicts excluded children from participating.

### Materials

An extensive interview scale was created and administered through individual interviews measuring the responses of 27 randomly selected adolescents living in homesteads or a hostel in southern Africa who were attending school in the same area and met the criteria of OVC. This questionnaire was intended not only to collect demographic data but also to establish rapport with the children and to obtain important information about their previous and current living experiences. From these interviews, a comprehensive self-report scale that was culturally sensitive was constructed to assess the well-being of individuals living in difficult settings who had lost at least one parent to HIV/AIDS. A culturally sensitive instrument has been called for in the literature for some time (Forehand et al., 1998) since the culturally relevant details of the lives of these OVC may be responsible for the conflicting results found across similar assessment tools in some studies (Forsyth, Damour, Nagler, & Adnopoz, 1996). These factors as well as the desire to more directly measure adolescent’s attachment behaviors and feelings of social support motivated the creation of the Psycho-Social Adjustment Scale-Adolescents (PAS-A). Adolescence for this particular study was defined from ages 12–24 since many of the children studied did not begin formal schooling until age 10–12. The PAS-A is comprised of 27 items that measure positive well-being and attachment as well as depression and anxiety. It also includes four critical items measuring hunger, safety, exposure to violence and suicide ideation, one item to measure social support (“I have adults I can trust”) and one item to measure attachment behavior (“Do you share your feelings when you are not happy”). Through the structured interviews it became apparent that these areas were of extreme importance in the children’s lives and were not part of other standardized scales.

#### Psychometric properties of the PAS-A

To assess levels of concurrent validity, two additional standard instruments commonly used with OVC samples were administered. The Reynolds Adolescent Depression Scale (RADS-2; Reynolds, 2002) short form, a 10-item standardized instrument, was used to measure depression. Validation of the RADS-2 has been established and reliability estimates are consistently high (*α*s* *>* *.80; Stark & Laurent, 2001). Anxiety was measured using the Revised Children’s Manifest Anxiety Scale (R-CMAS; Reynolds & Richmond, 1978) also a 10-item standardized scale with high reliability estimates (*α*s* *>* *.70; Holloway et al., 1958; Stark & Laurent, 2001). As expected, the PAS-A well-being scores negatively correlated with both the RADS depression scores (*r *= −.47) and R-CMAS anxiety scores (*r *= −.46); also, PAS-A depression and anxiety scores were positively correlated with both the RADS (*r *= .46) and R-CMAS (*r *= .47). Internal reliability estimates for the RADS (*α *= .76) and R-CMAS (*α *= .66) were both high with this current sample, but not as high as the corresponding subscale for depression and anxiety on the PAS-A (*α *= .81).

The PAS-A was also tested for psychometric properties using internal reliability coefficients and exploratory factor analysis. The original 48 items were created to measure positive well-being, attachment, anxiety, depression and parental ideation. Factor analysis yielded three general subscales using 27 items: well-being and attachment (11 items, *α *= .84), depression and anxiety (14 items, *α *= .81) and parental ideation (2 items, *α *= .58). Four critical items were identified that assess quality of life issues which include safety, suicide, hunger and violence, all significant attributes that contribute to the overall well-being of a child in this environment. As such, the PAS-A was seen to be a strong cohesive instrument as it measured depression and anxiety with higher reliability than both the RADS and R-CMAS and simultaneously offered a strong subscale for positive well-being and four critical items.

### Procedure

The study took place between March 2013 and February 2014. The authors’ Institutional Review Board (IRB) reviewed and approved all procedures (protocol number PSY-14–24). Written and informed consent was obtained from both the children and their designated caregiver. Prior to data collection, the first author and a translator visited each child’s family in their home where they explained the study and received written consent. In the event that the child was orphaned, consent was received from the caretaker assigned to that child at the NFPO. When beginning the interview with the child, the study was explained, and written consent was obtained for each participant. Prior to initial administration, all items were carefully examined by local translators to assure that the children (and translators) would understand the questions. Interviewers included the first author or a clinical psychologist who was trained for this project prior to administration. A translator trained in test administration accompanied all interviewers. Data were collected in the children’s school or living environment. During the interviews, the researcher read each question and the child responded by marking the box on the form that best described their feelings. A translator was present to assist if a child was unsure of a vocabulary word. Since most of the children were fluent in English very little support was required. Responses were then translated back to the researcher as the child marked the box on their paper. The interview and assessments lasted approximately 45 minutes and included completion of the instruments followed by a final in-depth interview guided by the responses during the assessment particularly if the child reported exposure to violence and/or suicide ideation.

Missing data were generated in one of the two ways. Firstly, during the interview some children may not have been able to complete all questions during the time allotted or may have declined to answer some questions. Secondly, data were collected in two waves. During the second wave, additional items were included on the PAS-A. As such, some analyses with individual items are based on this sub-sample.

## Results

Overall the sample’s scores for positive well-being and attachment were above the mid-point *M *= 2.99 [2.86, 3.12]. Scores for depression and anxiety were lower than the mid-point *M *= 1.89 [1.81, 1.97]. As illustrated in Table 1, when comparing these between living arrangements, children residing at the hostel had elevated but not significantly higher positive well-being and attachment scores, *t*(82.78) = −1.18, *p *= .24, *d *= 0.26. Depression and anxiety scores were similar between groups, *t*(87) = −0.172, *p *= .86, *d *= 0.04. There were also no significant differences on the four critical items between hostel and homestead children (see Table 1).

**Table 1.** Well-being, depression, anxiety and critical items between living arrangements.

	Overall (*N* = 89)	Homestead (*N* = 54)	Hostel (*N* = 35)	*t*	*p*	*d*
PAS-A, *M*(SD)						
Positive well-being and attachment	2.99 (0.66)	2.92 (0.73)	3.09 (0.58)	−1.183	.24	0.26
Depression and anxiety	1.89 (0.40)	1.87 (0.44)	1.88 (0.32)	−0.172	.86	0.04
Critical items (% yes)				*χ*^2^	*p*	*φ*
Safety	85%	80%	91%	2.23	.13	.15
Hunger	50%	57%	48%	0.44	.50	.09
Violence	72%	64%	83%	2.12	.14	.20
Suicide ideation	24%	24%	23%	0.01	.89	.01

The two specific items that asked about social support and attachment behavior were compared to positive well-being and attachment scores as well as depression and anxiety scores. There were significant positive correlations between positive well-being and attachment scores and social support, *r *= .44, *p* *< *.001, and attachment behavior, *r *= .30, *p *< .01. The correlations between these social support and attachment behavior and depression and anxiety scores were negative but small and non-significant (*r*s ≤ −.16). To further examine the potential impact of social support and attachment, the sample was grouped based on their responses to these two items. Two groups were formed for each consisting of those who responded “no” to the item and those who indicated some degree of agreement (“sometimes” – “almost all of the time”). Descriptive data for all comparisons are provided in Table 2. When comparing those with and without social support, the difference in positive well-being and attachment scores was marginally statistically significant but with a large effect size, *t*(59) = −1.99, *p *= .05, *d *= 0.51. Those with trusted adults had a higher positive well-being score, *M *= 3.16 (0.69), compared to those without trusted adults, *M *= 2.60 (1.01). There was a small non-significant effect of social support on depression and anxiety scores, *p *= .26, *d *= 0.29. When comparing children who do and do not exhibit attachment behaviors, there was a large significant effect on positive well-being, *t*(19.51) = −2.19, *p *= .04, *d *= 0.60. Those who shared their feelings had a higher average score of positive well-being and attachment *M *= 3.10 (0.57) while those who do not share their feelings had an average scores just above the mid-point *M *= 2.61 (0.87). There was no significant difference between these groups’ overall depression and anxiety scores, *p *= .88, *d *= 0.04.

**Table 2.** Well-being, depression, anxiety and critical items across social support and attachment behaviors.

	Share feelings				Have trusted adults			
	Yes	No	*t*	*d*	Yes	No	*t*	*d*
PAS-A, *M*(SD)								
Positive well-being and attachment	3.10 (0.57)	2.61 (0.87)	−2.19*	0.60	3.16 (0.69)	2.61 (1.01)	−1.99	0.51
Depression and anxiety	1.86 (0.36)	1.87 (0.46)	0.15	0.04	1.84 (0.47)	2.05 (0.60)	1.12	0.29
Critical items (% yes)			*χ*^2^	*φ*			*χ*^2^	*φ*
Safety	92.8%	58.8%	51.59***	.77	88.7	62.5	33.92***	.80
Hunger	43.9%	88.9%	3.84*	.38	49.1	62.5	14.22***	.67
Violence	70.7%	77.8%	13.44***	.61	69.8	87.5	20.44***	.68
Suicide ideation	17.9%	29.4%	5.00*	.50	15.1	62.5	0.69	.23

*p<.05; ***p<.001.

The groups were then compared on the critical factors, safety, hunger, violence and suicide. Frequencies for each group are provided in Table 2. When comparing those who do and do not exhibit attachment behavior, there were moderate-to-large significant differences across all four critical factors: safety, *χ* ^2^(1) = 51.59, *p *< .001, *ϕ *= .77; hunger, *χ* ^2^(1) = 3.84, *p *= .05, *ϕ *= .38; violence, *χ* ^2^(1) = 13.44, *p *< .001, *ϕ *= .61 and suicide, *χ* ^2^(1) = 5.00, *p *= .025, *ϕ *= .50. Those with and without social support showed large significant differences for safety, *χ* ^2^(1) = 33.92, *p *< .001, *ϕ *= .80, hunger, *χ* ^2^(1) = 14.22, *p *< .001, *ϕ *= .67 and violence, *χ* ^2^(1) = 20.44, *p *< .001, *ϕ *= .68, but not suicide, *p *= .40, *ϕ* = .23. Finally, the frequency of children who reported having social support was compared to the frequency of those exhibiting attachment behavior. Of children who reported having trusted adults, 87% reported sharing their feelings when not happy, *χ* ^2^(1) = 24.20, *p* **< .001, *ϕ *= .73.

## Discussion

This study investigated the role of social support and attachment on positive well-being among OVC children due to HIV/AIDS. This population is at increased risk for complicated bereavement given the increased number of psychological problems experienced by OVC in rural and economically deprived environments (Chi & Li, 2013). Both theoretical models and recent research suggest that attachment to caregivers is critical in helping these children through the bereavement process and life transitions. As such, we hypothesized that those children with access to trusting adults and exhibiting attachment behaviors would have higher positive well-being and lower rates of depression and anxiety.

No differences were found between the living arrangements of children (i.e., hostel vs. homesteads) regardless of hostel children coming from the most vulnerable situations. When comparing social support, defined as access to trusted adults, those with social support had significantly higher rates of positive well-being and attachment. Those who shared their feelings when not happy had higher positive well-being and attachment scores. Importantly, children who reported having trusting adults were significantly more likely to share their feelings when not happy. This might be most important given the fact that children who do not share their feelings have significantly higher rates of hunger, exposure to violence, feeling unsafe and suicide ideation.

While orphanhood is an important risk factor and previous research has paid close attention to this, other factors related to the psychosocial development and attachment of these children, regardless of orphanhood, are important. The ability to feel safe in one’s surroundings and share your feelings is critical for all OVC children to cope, and this has been continually supported by the data gathered for this study. While the data showed that overall the children in this study were doing well in general, they continued to live in high-risk settings. Approximately half of the children reported, to at least some extent, going to bed hungry and a high percentage report seeing people being harmed in their community. One of the advantages of using the PAS-A is that the critical items within the scale help to identify children who may be continuing to struggle with respect to safety, hunger, suicide ideation and violence. In general, children on homesteads have higher rates of going to bed hungry, suicidal ideation and feelings that they do not live in a safe place. Given these differences and the idea that living arrangements create important differences impacting the development of children, it was expected that access to trusted caregivers would be an important protective factor.

Examining the attachment of children living in both the hostel and homesteads made it clear that being connected to a trusted adult and sharing your feelings when not happy are related to the overall well-being. Children who have trusted adults and share feelings have higher levels of well-being and those who do not are more likely to report higher levels of hunger and exposure to violence. Regardless of their high-risk situations they still retain levels of well-being that are quite high. This indicates that they continue to thrive as illustrated by their high well-being and low depression and anxiety. This was interpreted as evidence that the environment of the hostel or homestead is allowing for appropriate emotional development and processing for children when trusting adults are present. When they attach to these caregivers they feel safe, cared for and happy and, as such, have low rates of depression and anxiety and high levels of well-being.

This study is not without limitations. The sample was drawn from a related community and consisted of only 100 children. Further, although there was a consistent relationship between positive outcomes and sharing feelings, the direction of this relationship could be such that children who are happier overall are more inclined to share unhappy feelings that they experience. Access to trusted adults was also related to more positive outcomes, and the direction of this would be less clear if the children who have difficult circumstances received more comprehensive care provided by the organization as this could lead to more access to trusted adults. In this particular sample, the children living in the hostile were not necessarily in more difficult situations making access to trusted adults the more likely precursor to positive outcomes. Collecting data from larger samples of children in these various living arrangements may allow for more detailed analysis regarding the direction of these relationships and potential confounders.

## Conclusion

This study ends with grave concern and recommendations for addressing these concerns. Due to the instability and lack of “parents” and parental figures as a result of HIV/AIDS pandemic, we need to recognize that in order for this group to emerge into adulthood, social institutions (e.g., government and child welfare) need to recognize the need for extended education time lines, health care and psychosocial services. Failing to protect and support vulnerable youth at a critical time would result in significant downward spiraling destroying the very fabric of their fragile culture. A new urgency is needed to support and mentor this group who may in cases do not have adult attachments to support and mentor them into adulthood. Trusting relationships are paramount to healthy psychological recovery. Children need to have an outlet to share their unhappy feelings.

The administration of the PAS-A is a starting point to measure the well-being of children in this vulnerable population. Through its administration one can measure depression, anxiety and attachment and attachment with a trusted individual, which appears by this current work to be essential in the development of well-being.

This research identified the role of attachment as it impacts the psychological well-being of children who are impacted by the debilitating conditions related to HIV/AIDS in southern Africa. Further studies using standardized instruments are needed to continue to explore the role of attachment among children who are in extreme psychological distress. From this initial study, it has become clear that being connected to a trusted adult and sharing feelings when one is not happy are important in establishing positive well-being in children impacted by HIV/AIDS in southern Africa.
